# Failure to detect tuberculosis in Black lechwe antelopes (*Kobus leche smithemani*) in Zambia

**DOI:** 10.1186/1756-0500-4-233

**Published:** 2011-07-05

**Authors:** Musso Munyeme, John B Muma, Hetron M Munang'andu, King S Nalubamba, Clovice Kankya, Eystein Skjerve, Jacques Godfroid, Morten Tryland

**Affiliations:** 1 Department of Disease Control, The University of Zambia, School of Veterinary Medicine, P.O. Box 32379 Lusaka, Zambia; 2 Department of Basic Sciences and Aquatic Medicine, Section of Aquatic Medicine and Nutrition, Norwegian School of Veterinary Science, P.O. Box 8146 Dep., 0033 Oslo, Norway; 3 Department of Clinical Studies, The University of Zambia, School of Veterinary Medicine, P.O. Box 32379 Lusaka, Zambia; 4 Department of Food Safety and Infection Biology, Norwegian School of Veterinary Science, P.O. Box 8146 Dep., 0033 Oslo, Norway; 5 Department of Food Safety and Infection Biology, Section of Arctic Veterinary Medicine, Norwegian School of Veterinary Science, Stakkevollveien 23, N-9010 Tromsø, Norway

## Abstract

**Background:**

Two types of lechwe antelopes exclusively exist in their natural ecosystems in Zambia; the Black lechwe (*Kobus leche smithemani*) and the Kafue lechwe (*Kobus leche kafuensis*). Despite inhabiting similar ecosystems, tuberculosis has been reported in Kafue lechwe without its documentation in Black lechwe antelopes. However, the past few decades have seen a drastic decline in both lechwe populations. Whereas studies have postulated that infectious diseases such as tuberculosis are having a negative impact on the Kafue lechwe population, no information is available on Black lechwe antelopes. Thus this study was conducted to investigate tuberculosis in Black lechwe antelopes of the Bangweulu swamps in comparison with the Kafue lechwe antelopes of Lochinvar.

**Findings:**

A total of 44 lechwe antelopes (Black (*n *= 30): Kafue (*n *= 14) were sampled from Bangweulu and Lochinvar respectively. A positive case was defined with findings of gross lesions with Ziehl Nielsen and culture confirmation. Out of the 14 animals examined in Lochinvar, 21.4% [95% CI: 15.4, 44.4%] had necropsy lesions consistent with tuberculosis. The corresponding samples from 30 Black lechwe of Bangweulu yielded negative results on all the three tests.

**Conclusions:**

Current findings from this study intimate the possible absence of tuberculosis in Black lechwe antelopes whilst confirming the presence of tuberculosis in Kafue lechwe of the Kafue basin. The absence of tuberculosis in the Black lechwe suggests that the observed population decline may not be caused by tuberculosis. However, without detailed molecular epidemiological studies it is not possible to determine the association of *M. bovis *infection in sympatric animal populations. The possible role of transmission of tuberculosis between wildlife and cattle is discussed herein. **Findings**

## Findings

## Background

Bovine tuberculosis has emerged as a serious threat to conservation wildlife [1-4]. In Africa the disease has been reported in several wildlife species [2,3]. It is endemic in African buffaloes (*Syncerus caffer*) in the Kruger and Ruwenzori National Parks and in Kafue lechwe antelopes (*Kobus leche kafuensis*) of the Kafue basin [1,3,4]. As pointed out by Bengis and others [5], the African buffalo and the Kafue lechwe antelopes have become true sylvatic wildlife maintenance hosts with sporadic spill over to other species. The earliest reports of bovine tuberculosis in the Kafue lechwe date back to the 1940s [6]. Circumstantial evidence indicates that the disease could have been introduced from cattle to lechwe through the practice of transhumance grazing in the Kafue basin [7-9].

Apart from the Kafue lechwe, Zambia is endowed with the Red lechwe (*Kobus leche Leche*) which is also found in Botswana and predominantly in the Liuwa plains of Western Province and the Black lechwe (*Kobus leche smithemani*) inhabiting the Bangweulu plains of northern Zambia. All *Kobus leche *subspecies are semi-aquatic medium sized antelopes that live in wetlands areas. They live in herds that increase in size during the rainy season when flood waters accumulate and during lekking in the breeding season when territorial males occupy small discrete areas clustered by groups of females that go for mating. These characteristics are common for all *Kobus leche *antelopes indicating that these animals have similar population structures besides inhabiting similar ecosystems comprising of wetlands and marsh areas [10,11]. As pointed out elsewhere, gregarious species that cluster in large numbers have the potential to maintain bovine tuberculosis within herds for a long time with occasional spillover to other species [12]. In some areas, the wetlands inhabited by the *Kobus leche *antelopes are also used by livestock as grazing pastures leading to interspecies transmission of animal diseases between wildlife and livestock as commonly observed in the Kafue basin [13-15]. It has been assumed that the persistence of bovine tuberculosis could have contributed to the decrease in Kafue lechwe population of the Kafue basin leading to their subsequent inclusion on the International Union of Conservation (IUCN) red list of endangered species [10,16]. However, the decrease in population is across all lechwe species.

The absence of a livestock/wildlife interface area in the presence of one of the three lechwe species in the Bangweulu swamps provided a unique and important epidemiological determinant in assessing the maintenance of tuberculosis in wildlife and cattle in Zambia given that the Kafue lechwe of the Kafue basin have been shown to be a reservoir host of tuberculosis [3,11,16,17].

The key explanatory difference between the Kafue basin and the Bangweulu swamps is the absence of a livestock/wildlife interface area in the latter. As for the Black lechwe, there is marked paucity of information with regards to tuberculosis. Information obtained on the ground during the sampling period from Zambia Wildlife Authority (ZAWA) and seasonal Safari hunters indicated that they have never come across any suspected case of tuberculosis in the Black lechwe antelopes of the Bangweulu swamps. However, ZAWA officials, cattle owners and Safari hunters in the Kafue basin are very much aware of the presence of tuberculosis in the Kafue lechwe antelopes [15]. Further, in the Kafue basin, tuberculosis prevalence is estimated at 27.7% in Kafue lechwe antelopes [3]. In cattle, tuberculosis prevalence is estimated at 9.6% in the blue lagoon area whilst in Lochinvar it's estimated at 5.2% [13]. Epidemiological models and baseline studies conducted on the prevalence of bovine tuberculosis within Zambia indicate a foci of infection and a relatively high prevalence level in the Kafue basin [7,13,14]. This focus of tuberculosis is probably due to active and continuous disease transmission between and within species [12]. The grazing range of Kafue lechwe and cattle extensively overlap and during the dry season the frequency of interaction increases especially at watering points and in areas with green pastures. This scenario increases the opportunity for interspecies transmission of tuberculosis. The pathology and epidemiology of the disease in cattle and lechwe is similar and respiratory in nature [12,18]. However, to date, no structured studies or documentation of tuberculosis are available on Black lechwe antelopes from the Bangweulu plains.

ZAWA, an organization mandated by government to restore natural environments and ecological communities that inhabit them have for a long time recorded and reported a continuous decline in the *Kobus leche *populations in Zambia [11,19]. The Black lechwe population on the Bangweulu plains declined from approximately 150,000 animals in 1930s to about 30,000 in the 1980s while the Kafue lechwe has decreased by 85% in the last 75 years from 250,000 animals in 1931 to 38,000 in 2005 [20] leading to their subsequent inclusion on the IUCN red list of endangered species [21]. However, current population censuses of the Black lechwe indicate slight recovery in numbers as a result of a deliberate policy by ZAWA utilizing light off-take rates able to sustain a positive population recovery.

Although studies have shown that animal diseases inclusive of bovine tuberculosis have significantly contributed to the reduction of the Kafue lechwe population on the Kafue basin, factors leading to the reduction of the Black lechwe population on the Bangweulu plains are yet to be elucidated. Hence, ZAWA through its research unit has embarked on carrying out investigations aimed at determining causal factors that have the potential to cause extinction of the *Kobus leche *populations in Zambia. These investigations are expected to determine, among other things, ecological factors that allow for the introduction of exotic diseases into both sympatric and allopatric wildlife populations, assessing the impact of these diseases on conservation and identifying mitigation measures aimed at preventing and or reducing the occurrence of animal diseases in wildlife populations. In this present study, the objective was to determine if the Black lechwe found in the Bangweulu swamps an area predominantly occupied by wildlife without the presence of livestock are infected with tuberculosis. Thus livestock/wildlife interaction in disease transmission was assessed using a similar wildlife population that has no contact with wildlife to elucidate the effect of this interaction.

## Results

A total of 44 *Kobus leche *antelopes (Black lechwe (*n *= 30) and Kafue lechwe (*n *= 14)) (Table 1), were sampled from the Bangweulu and the Lochinvar Game Management areas between October to November 2009. Age ranges overlapped between the two sampling strata with the Lochinvar lechwe having an average age of 11.5 years (95% CI; 9.5 to 13.5 years), whilst in Bangweulu, the average age was 8.9 years (95% CI; 7.7 to 10 years).

**Table 1:** Tuberculosis survey results in Black and Kafue lechwe antelopes conducted between October to November 2009

Study Area Factors Under Consideration	Kafue Basin	Bangweulu swamps	Overall Totals across both Strata
No of animals sampled	(*n *= 14)	(*n *= 30)	(*n *= 44)
Cattle	Present **(+)**	NONE **(-)**	-
Livestock/wildlife interface	Present **(+)**	NONE **(-)**	-
Positive Necropsy	3 (21.4%)	0	3 (6.8%)
	[95% CI: 15.4, 44.4%]	-	[95% CI: 0, 14.1%]
ZN positive	4 (28.6%)	0	4 (9.1%)
	[95% CI: 3.2, 53.8%]	-	[95% CI: 1.0, 17.1%]
Positive Culture	4 (28.6%)	0	4 (9.1%)
	[95% CI: 3.2, 53.8%]	-	[95% CI: 1.0, 17.1%]

None of the 30 Black lechwe sampled showed gross post-mortem lesions that were suggestive of tuberculosis. When these samples were subjected to Ziehl-Neelsen, there were no observable acid fast bacteria on microscopic examination. We failed to cultivate the bacteria from any sample that came from Bangweulu, yet four characteristically tuberculosis culture colonies grew out of the 11 Kafue lechwe sampled from the Lochinvar Game Management Area (Table 1).

Of the three Kafue lechwe antelopes that were positive on necropsy results, none had a poor body condition score. One animal, positive on both Ziehl-Neelsen and culture but without visible lesions had a poor body condition score.

A positive case was defined with findings of gross lesions with Ziehl Nielsen (ZN) and culture confirmation. Out of 14 animals examined in the Lochinvar Game Management Area of the Kafue basin, 21.4% [95% CI: 15.4, 44.4%] of the lechwe carcasses had necropsy lesions suggestive of tuberculosis; with 28.6% [95% CI: 3.2, 53.8%] of the tissues (4/14) indicating acid fast staining (ZN) bacteria as well as culture and morphological characteristics suggestive of mycobacteria (Table 1).

## Discussion

This is the first epidemiological study in Black lechwe of the Bangweulu swamps demonstrating the absence of tuberculosis. Notwithstanding the small sample size of antelopes from the Kafue basin, these results do not deviate from earlier epidemiological findings from studies that demonstrated the existence of tuberculosis in Kafue lechwe antelopes [3,22]. Limitations in sample size in wildlife studies are not uncommon especially with rare wildlife species threatened with extinction; nonetheless, our results are a valid assessment and representation of the epidemiological situation with regards to tuberculosis in both the Kafue basin and the Bangweulu swamps. Specifically, this being the first study to systematically analyse results suggesting the absence of tuberculosis in the Black lechwe of the Bangweulu swamps were cattle are absent, intimating on cattle to be a vital deterministic factor in tuberculosis transmission with wildlife.

**Figure 1:**
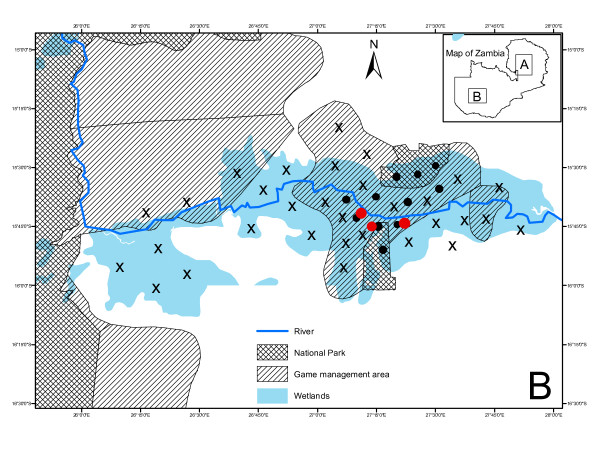
**Map of the Kafue basin showing the interaction between cattle, black crosses (X), and the Kafue lechwe antelopes (black dots)**. Sampling sites are indicated as red dots. Insert is Map of Zambia (**A**, Being Bangweulu swamps whilst **B **is the Kafue Basin)

Reports based on meat inspection at the Bangweulu area command unit of ZAWA obtained from safari hunting also indicate the absence of bovine tuberculosis in the Bangweulu swamps. Whereas similar reports of meat inspection on animals obtained from safari hunting and epidemiological surveys carried out in the Kafue basin indicate the presence of the disease. With the Kafue lechwe being the most sought after wildlife species utilized for game meat consumption [10] these findings pose a significant public health threat to the general public. Our observations further indicate that the close interaction between cattle and the Kafue lechwe (Figure 1) is likely to enhance sharing of diseases between the two animal species and this is in concordance with earlier findings [16,22,23]. Hence, inter-species transmission of bovine tuberculosis and other diseases between cattle and Kafue lechwe could be an ongoing process although it is not yet clear which of the two animal species maintains the infection in the ecosystem [3,24].

Despite the tuberculosis situation in the Kafue basin having been well documented [3,8,14,22], the direction of transmission between cattle and lechwe is yet to be elucidated as available evidence is still patchy. On the other hand, results from our present study have confirmed earlier reports of tuberculosis in the Kafue basin where the disease has become established and well maintained and not in the Bangweulu swamps ecosystem. Further the Kafue basin scenario has been different from the Bangweulu swamps from as early as the 1900s when European Colonial settlers drove large herds of cattle onto the Kafue basin. Both Lochinvar and Blue Lagoon National Parks in the Kafue basin were only gazetted as protected areas and transformed into National Parks in early 1970s. Prior, they were extensive cattle ranches for the European settlers [23], a clear distinction from the Bangweulu swamps which have never been cattle ranches.. Thus the early introduction of livestock to the Kafue basin strongly augments the postulation that cattle may have been the source of infection for the Kafue lechwe antelopes. This study therefore forms part of a larger study to describe the epidemiology of tuberculosis in the Kafue lechwe antelopes in Zambia by providing baseline epidemiological data for future molecular work.

These findings indicate that bovine tuberculosis might not be linked to the reduction of the Black lechwe population in the Bangweulu swamps. Both lechwe antelope species are particularly vulnerable to high poaching rates, a factor that needs detailed assessment on its effect on population decline. In the Kafue basin, unlike in the Bangweulu swamps, high human settlement pressure coupled with increasing grazing pressure on few available pasturelands is another factor affecting population stability.

Information obtained from our study is very important for future management decisions when introducing wildlife or livestock from areas endemic with bovine tuberculosis to the Bangweulu ecosystem. Considerations aimed at avoiding introducing bovine tuberculosis in the Bangweulu ecosystem have to be formulated. On the other hand, the translocation of wildlife from *ex-situ *conservancies to game ranches demands that only animals free of highly infectious diseases such as bovine tuberculosis have to be translocated to game ranches. Our findings indicate that Black lechwe from the Bangweulu swamps would be good candidates for *in-situ *conservation given the absence of tuberculosis in their natural ecosystems.

## Conclusions

Current findings from this study intimate the possible absence of tuberculosis in Black lechwe antelopes whilst confirming the presence of tuberculosis in Kafue lechwe. The absence of tuberculosis in the Black lechwe suggests that the observed population decline may not be caused by tuberculosis. However, without detailed molecular epidemiological studies it is not possible to determine the association of *M. bovis *infection in sympatric animal populations. The results further intimate on the possible role of cattle in the transmission of tuberculosis to wildlife in the Kafue basin.

## Methods

### Ethical approval

Approval to conduct this study in both the Bangweulu and Lochinvar Game management areas was given by both the Research Unit of the Zambia Wildlife Authority (ZAWA), and the Board of Post Graduate Studies and Research of the School of Veterinary Medicine at the University of Zambia.

### Study areas

**Figure 2:**
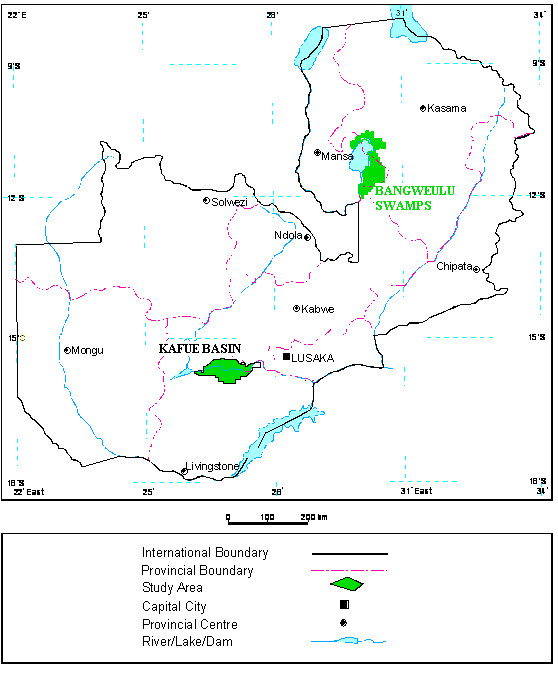
**General location of the two study sites within Zambia**.

Two wetlands were selected for this study, namely the Bangweulu swamps inhabited by the Black lechwe antelopes and the Kafue basin inhabited by the Kafue lechwe antelopes (Figure 2).

The Kafue basin (Figure 1) is a sprawling floodplain covering an area of about 6,000 km^2 ^[10,25,26] comprising Lochinvar (410 km^2^), Blue Lagoon National Park (420 km^2^) and the Game Management Areas (GMAs) (5,175 km^2^) [27]. The interface areas of the Kafue basin National Parks are endowed with wildlife, particularly the Kafue lechwe antelope which interacts freely and easily with livestock (cattle) (Figure 1). Ecological details of the Kafue basin are fully described elsewhere [3,10,25,26]. Like the Bangweulu ecosystem, the Kafue basin GMA is overlaid by wetlands (Figure 1). The principal activity in the Kafue basin is the transhumance grazing system practiced by the local inhabitants. The wetlands provide grazing pastures to more than 300,000 herds of cattle (Figure 1) that come to the wetlands during the dry season. Physical inspection of the study areas showed presence of livestock, cattle trail routes as well as cattle-dung deep inside the Lochinvar National Park and the Game Management Areas. Besides, cattle were present in the study area during the time of sample collection. Figure 1 shows cattle and Kafue lechwe grazing areas as geo-referenced by the ZAWA officials.

**Figure 3:**
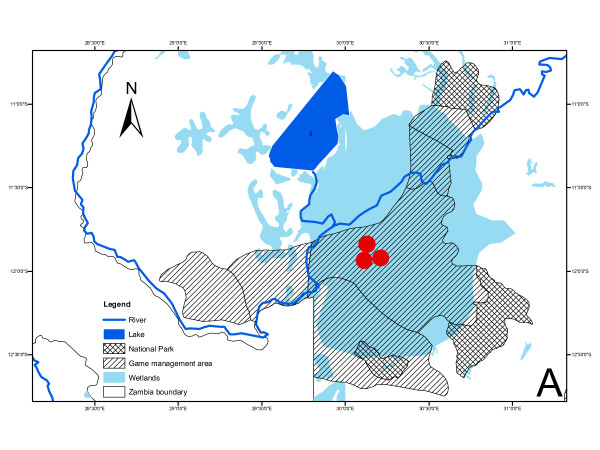
**Map of the Bangweulu swamps with sampling sites indicated as red dots**.

The Bangweulu swamps (Figure 3) are a recognised and important Ramsar Site 531 and as such the area is a protected ecosystem [28]. The area is a complex sprawling floodplain that stretches into the horizon in the remote northern reaches of Zambia (Figure 2) covering a portion of Isangano National Park to the north-east, Bangweulu Game Management Area (GMA) number 26 in the centre, Chambeshi GMA Number 27 to the East, Kafinda GMA to the south, Lavushi Manda National Park south-east and Mansa GMA Number 31 to the south-west. Geographically, the swamps are located by coordinates 10° 33' S, 029° 15' E and 12° 17' S, 030°43' E with an elevation of between 900 to 1200 m above sea level, covering an area of approximately 31,000 square kilometres. The Bangweulu ecosystem comprises of lakes (Figure 3), swamplands, seasonally flooded grasslands and innumerable shallow water bodies linked by an intricate network of channels. Dry land is also found in the form of termitaria that occurs within the seasonally flooded areas providing further diversity to the area. It supports large numbers of the endemic, semi-aquatic Black Lechwe, vulnerable on IUCN list of threatened species. It is home to the threatened wattled crane (*Grus carunculatus*) and provides the only existing habitat in Zambia for the threatened shoebill stork (*Balaeniceps rex*) [21]. Unlike the Kafue basin which is a pastoralist area, the principle activity in the Bangweulu swamps is fishing being the largest fishing area in Zambia. Historically, there are no records of cattle rearing in the wetlands and no transhumance grazing system is practiced. Physical inspection of the sampling sites showed no signs of livestock or livestock-dung and reports obtained from the ZAWA and Veterinary Officers confirmed the absence of livestock in the area.

### Designing the Study

Our study was based on the animals that were allocated for the research quota for 2009 by the Zambia Wildlife Authority (ZAWA). Given the endangered status of the *Kobus leche *antelopes in Zambia, only 30 Black lechwe antelopes and 14 Kafue lechwe antelopes were allocated for the study. Although the sample size was dependent on the number of animals allocated for the study, selection of the study herds and individual animals was done at random.

### Sample collection

Lechwe harvested for necropsy had their ages estimated by utilizing the ring patterns on the horns and tooth development and wear patterns. Detailed post-mortem examination of the carcasses was done as described elsewhere [29]. Data pertaining to each individual animal was gathered, and this included information on age, sex, body condition score, parasite burden, area from which the hunting was conducted by Geo-referencing each site where the animals where harvested from. Body condition was scored according to the amount of fat, and each carcass was allocated a score of 1 (poor: almost no fat); 2 (fair: average fat amount present); or 3 (good: plentiful fat, completely obscuring the kidneys). Field assessment of the kidney fat index was also undertaken. The muscle conditions were also used to assist in scoring the general nutritional body status for coming up with an average body condition score of the carcass.

### Decontamination and Culturing

All the TB suspect tissue and organs were decontaminated in the Biohazard Safety Cabinet (Bio-safety Level 2) in the laboratory with the procedure having been described elsewhere [3,11,16,17].

### Ziehl-Neelsen staining

All the slide preparates of smears from decontaminated tissue homogenates were stained as previously described [3,11,16,17].

### Direct microscopic smear examination

All the stained slides were subjected to a direct microscopic examination under oil immersion. Interpretation was based on the recommendations stipulated by OIE tabulations as well as the 'American Thoracic Society' scale from Lenneth adapted at the Chest Disease Laboratories (CDL)-Lusaka Zambia. The interpretation scales involved examination of 200 fields. The Ziehl-Neelsen staining procedure was used for microscopic examination.

### Data Storage and Statistical analyses

The database was established and stored in Microsoft^® ^Excel spread sheets before transferring data to Stata SE/10 for Windows (Stata Corp. College Station, TX, USA). The database included information about sex, age, parasite burden, area of study and body condition score at animal level. Area level data included information about ecological factors. Cattle interaction etc. Crude estimates for tuberculosis proportions were computed using the survey command estimates in Stata with adjustments for strata (study area) as described by Dohoo and coworkers.

## Competing interests

The authors declare that they have no competing interests.

## Authors' contributions

MM contributed to the design, sample and data collection and analysis and drafting of the manuscript; JBM contributed to sampling and supervision of the field work and writing of manuscript; HMM contributed to drafting, generating the maps and preparation of the manuscript; KN contributed to field study design, sample collection, manuscript editing and important intellectual contribution; CK contributed to drafting and reviewing of the manuscript; ES contributed to conception, design and the writing of manuscript, JG contributed to writing of the manuscript and important intellectual contribution, MT contributed to supervision of the project, acquisition of parts of the funds and writing of the manuscript and important intellectual contribution. All authors have read and approved the final manuscript.
